# Antimicrobial effects of combined piezoelectric cold plasma, organic acids, and nanoemulsions against *Salmonella* Typhimurium and *Listeria monocytogenes* on pork

**DOI:** 10.1007/s00253-026-13739-8

**Published:** 2026-02-09

**Authors:** Yelyzaveta K. Oliinychenko, Brijesh Tiwari, Alexandros Ch. Stratakos

**Affiliations:** 1 Centre for Sustainable Agri-Food and Environment (SAFE), School of Applied Sciences, College for Health, Science and Society, University of the West of England, Coldharbour Lane, Bristo, BS16 1QY UK; 2 Department of Food Biosciences, Teagasc Food Research Centre, Dublin 15, Teagasc, D15 DY05 Ashtown Ireland

**Keywords:** Cold plasma, Foodborne pathogens, Meat decontamination, Meat safety, Non-thermal processing, Sustainable food preservation

## Abstract

**Abstract:**

This study evaluated the antimicrobial efficacy of cold atmospheric plasma (CAP) alone and in combination with nanoemulsions or organic acids against pathogens on polycarbonate membranes and pork meat. CAP was generated via piezoelectric direct discharge technology with ambient air as the working gas. On polycarbonate membranes, CAP treatment alone for 15, 30, and 45 s reduced *Salmonella *Typhimurium by 0.9, 1.4, and 2.4 log CFU/cm^2^ and *Listeria monocytogenes* by 0.7, 1.7, and 2.3 log CFU/cm^2^, respectively, showing a time-dependent antimicrobial effect. When CAP was applied before lactic or acetic acid (at minimum inhibitory concentrations (MICs)/minimum bactericidal concentrations (MBCs)) on polycarbonate membranes, the combined treatments achieved significantly greater reductions (~ 3.6 log CFU/cm^2^) than when acids were applied before CAP (~ 2.3 log CFU/cm^2^), highlighting the importance of application sequence. Overall, CAP treatments on polycarbonate membranes showed additive effects when CAP (applied for 15, 30, or 45 s) was combined with antimicrobials (at MIC/MBC). On pork, CAP treatment for 9 min combined with organic acids or nanoemulsions at 10× MIC produced significant additive effects, enhancing pathogen inactivation (by ~ 1.5 log CFU/g) compared with CAP alone or antimicrobials alone under the same conditions. These findings support the application of CAP–antimicrobial combinations as a non-thermal, sustainable strategy to improve meat safety. Further research should evaluate the impact of treatments on the sensory attributes of meat and support their implementation at an industrial scale.

**Key Points:**

•*Cold atmospheric plasma (CAP) generated via piezoelectric direct discharge significantly reduced pathogens on pork and membranes.*

•* CAP efficiency against Salmonella Typhimurium and Listeria monocytogenes significantly increased with extended exposure time.*

•*Applying CAP prior to organic acids, significantly increased antimicrobial efficacy, confirming sequence effects.*

•*CAP combined with antimicrobials reduced pathogens on pork by ~1.5 log CFU/g.*

•*CAP–antimicrobial combinations represent a promising strategy for meat safety.*

**Graphical Abstract:**

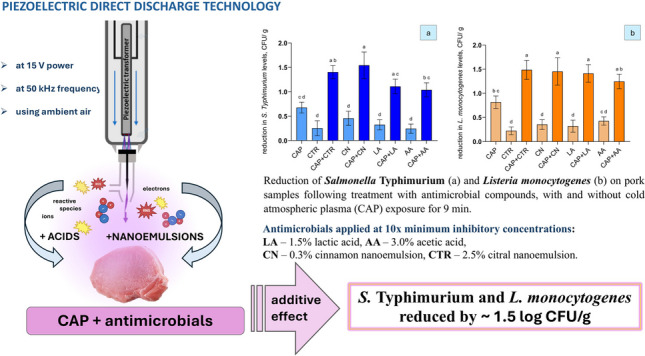

## Introduction

Meat safety remains a critical concern, particularly in the context of evolving production methods, advanced processing techniques, and shifting consumer preferences for minimally processed foods and environmentally sustainable products (Ponnampalam & Holman 2023). *Salmonella *(*S*.) spp. and *Listeria *(*L*.) *monocytogenes* are among the most prevalent pathogens associated with foodborne illnesses linked to meat consumption, posing significant risks to public health and food safety (Lianou et al. 2017). Between 1991 and 2021, pork consumption was implicated in 555 outbreaks globally, resulting in 14,977 reported illnesses and 4727 fatalities, with *Salmonella* spp. and *L. monocytogenes* accounting for 469 and 120 outbreaks, respectively (Warmate & Onarinde 2023). These alarming statistics underscore the necessity for effective interventions to mitigate microbial risks in pork production and consumption chains.

Conventional meat processing methods, such as heat treatment and the use of chemical additives, have proven effective in ensuring meat safety (Yu et al. 2021). However, these approaches often involve increased costs and compromise the nutritional quality and sensory attributes of meat and may not be in line with consumer demands (Gómez et al. 2020). Recent advancements in meat processing technologies have prioritised non-thermal methods, shorter processing times, cost-effectiveness, and environmental sustainability (Safwa et al., 2024; Lisboa et al., 2024). Cold atmospheric plasma (CAP) is a novel non-thermal and environmentally sustainable technology that effectively inactivates pathogens while meeting consumer demands for safer, minimally processed, and chemical-free food products (González-González et al. 2021; Jayasena et al. 2023).

CAP is described as the fourth state of matter, containing reactive oxygen and nitrogen species (ROS/RNS), charged particles, and ultraviolet (UV) radiation, which collectively contribute to antimicrobial efficacy (Jayasena et al. 2023). Various plasma sources, such as plasma jets, corona discharge, dielectric barrier discharge (DBD), and piezoelectric direct discharge (PDD), have been explored for their antimicrobial efficacy in meat processing (Jayasena et al. 2023; Oliinychenko et al. 2024). Previous research has shown that reactive species generated by CAP inactivate bacteria by disrupting cell walls, oxidising intracellular components, and inducing oxidative stress, which leads to DNA damage and impaired cell replication (Gan et al. 2021; Zhang et al. 2023).

Nanoemulsions (NEs) containing plant-derived antimicrobials enhance the delivery and efficacy of oil-based compounds in food applications, while organic acids, such as lactic and acetic acid, effectively reduce microbial contamination on food surfaces (Nkosi et al. 2021). The combination of CAP technology with plant-derived antimicrobials has demonstrated strong additive effects in microbial inactivation, particularly in meat products (Cui et al. 2017; Yoo et al. 2021). For instance, combining CAP (DBD, 2 min) with 0.5% lemongrass oil resulted in a 2.8 log CFU reduction of *Listeria monocytogenes* on pork loin, while CAP (DBD, 4 min) with 0.05% clove oil led to a 7.5 log CFU/g reduction of *Escherichia coli* on beef jerky (Cui et al. 2017; Yoo et al. 2021).

However, despite evidence of the antibacterial properties of CAP and its effects with antimicrobials in in vitro systems, the application of piezoelectric direct discharge CAP technology in meat processing and especially pork remains unexplored. In particular, its combined efficacy with nanoemulsions and organic acids on meat matrices has not yet been investigated. Addressing this gap is essential for evaluating the potential of piezoelectric direct discharge cold atmospheric plasma (PDD-CAP) for industrial-scale application.

The current study investigates the potential of CAP treatment, produced using PDD technology, in combination with antimicrobials, to enhance the safety of pork meat. Specifically, it evaluates the antimicrobial efficacy of organic acids or nanoemulsions loaded with essential oils (EOs), both individually and in combination with CAP, against *Salmonella* Typhimurium and *Listeria monocytogenes*. Initial testing on polycarbonate membranes was used to determine the most effective sequence of application, which was subsequently applied to pork meat.

## Materials and methods

### Preparation of bacterial inoculum

Two reference strains, *Salmonella enterica* subsp. *enterica* serovar Typhimurium (NCTC 112.19) and *Listeria monocytogenes* (WDCM 00021), were used in this study. Frozen stock cultures were stored at −80 °C until use.

To revive the bacteria, frozen stock cultures of *S.* Typhimurium and *L. monocytogenes* were streaked onto Tryptone Soya Agar (TSA; Oxoid, UK). The plates were then incubated at 37 °C for 24 h to allow the growth of isolated colonies. One isolated colony from each plate was aseptically transferred to 10 mL of Brain Heart Infusion Broth (BHI; Oxoid, UK) and incubated at 37 °C for 24 h. Following incubation, the pathogen cultures were centrifuged at 6500×*g* for 10 min to pellet the bacterial cells, and the supernatant was discarded. The resulting cell pellets were resuspended in 10 mL of Maximum Recovery Diluent (MRD; Oxoid, UK) and adjusted to an optical density of 1.0 at 600 nm using a spectrophotometer (Keison Products, Essex, UK) for further use. These standardised suspensions were used as the inoculum for subsequent experiments.

### Preparation and characterisation antimicrobial compounds

#### Preparation of lactic and acetic acid solutions

Lactic acid (LA) (Merck, UK) and acetic acid (AA) (Merck, UK) were diluted in sterile distilled water to prepare solutions at the required concentrations for subsequent experiments.

#### Preparation of citral and cinnamon nanoemulsions

Nanoemulsions were prepared following the protocols of Prakash et al. (2020) and Yang et al. (2023) with slight modifications. Citral (Merck, UK) or cinnamon oil (Merck, UK) was mixed with Tween 80 (Merck, UK) at a ratio of 1:2 and stirred at room temperature (20–25 °C) for 10 min at 1000 rpm using a vortex mixer (Thermo Scientific Vortex Mixer, Thermo Fisher Scientific, Waltham, MA, USA). Sterile deionised water was then added at a ratio of 1:7, and the mixture was stirred for an additional 30 min to form the initial emulsion. The resulting emulsion was then processed ultrasonically for 10 min in an ultrasonic bath (Clifton 1-Litre Heated and Timed Ultrasonic Bath; Nickel-Electro Ltd., Somerset, UK) to obtain the final nanoemulsion.

#### Physicochemical characterisation of nanoemulsions

The stability properties of citral (CTR) and cinnamon (CN) nanoemulsions, including particle size, zeta potential (ZP), and polydispersity index (PDI), were characterised using a Zetasizer Nano series ZS (Malvern Instruments Ltd., Malvern, UK) and disposable 1.5-mL cuvettes (Merck, UK), following the method described by Ghodke et al. (2023) and Arkoumanis et al. (2019). Measurements were performed on the day of preparation to ensure accuracy and consistency. The nanoemulsions were diluted 1:100 in distilled water and filtered through a 20-µm filter (Millipore, Merck, UK) to remove potential aggregates before analysis. To ensure precise characterisation, zeta potential measurements were conducted at a fixed scattering angle of 137°, while particle size and PDI were assessed using dynamic light scattering (DLS) under standard instrument settings.

### Determination of minimum inhibitory and bactericidal concentrations (MIC/MBC)

The minimum inhibitory concentrations (MICs) of CTR and CN nanoemulsions, as well as selected organic acids, were determined against *L. monocytogenes* and *S.* Typhimurium using the broth microdilution method described by Parvekar et al. (2020). Pathogen cultures of *L. monocytogenes* and *S.* Typhimurium were grown overnight in Mueller–Hinton broth (MHB; Oxoid, CM0405B, UK) at 37 °C under static conditions. The overnight pathogen cultures were diluted to an initial inoculum concentration of approximately 5 × 10^5^ CFU/mL.

Each well of the 96-well microplate contained 100 µL of the active compound at varying concentrations and 100 µL of bacterial inoculum. Negative controls consisted of wells containing inoculated broth without the active compounds. The plates were incubated at 37 °C for 24 h under static conditions. The MIC was defined as the lowest concentration of the test compound that prevented visible bacterial growth, as assessed by visual inspection of wells before and after incubation relative to control wells.

For minimum bactericidal concentration (MBC) evaluation, aliquots of 10 µL from wells showing no visible growth were plated on Tryptone Soya Agar (TSA; Oxoid, UK), a non-selective medium used for bacterial recovery and incubated at 37 °C for 24 h (Parvekar et al. 2020). The MBC was defined as the lowest concentration of nanoemulsions or acids that achieved a ≥ 99.9% reduction in the initial bacterial inoculum. The MBC endpoint was confirmed by assessing bacterial growth on the agar plates, with visible colonies indicating surviving bacterial cells.

### Experimental design

**Fig. 1:**
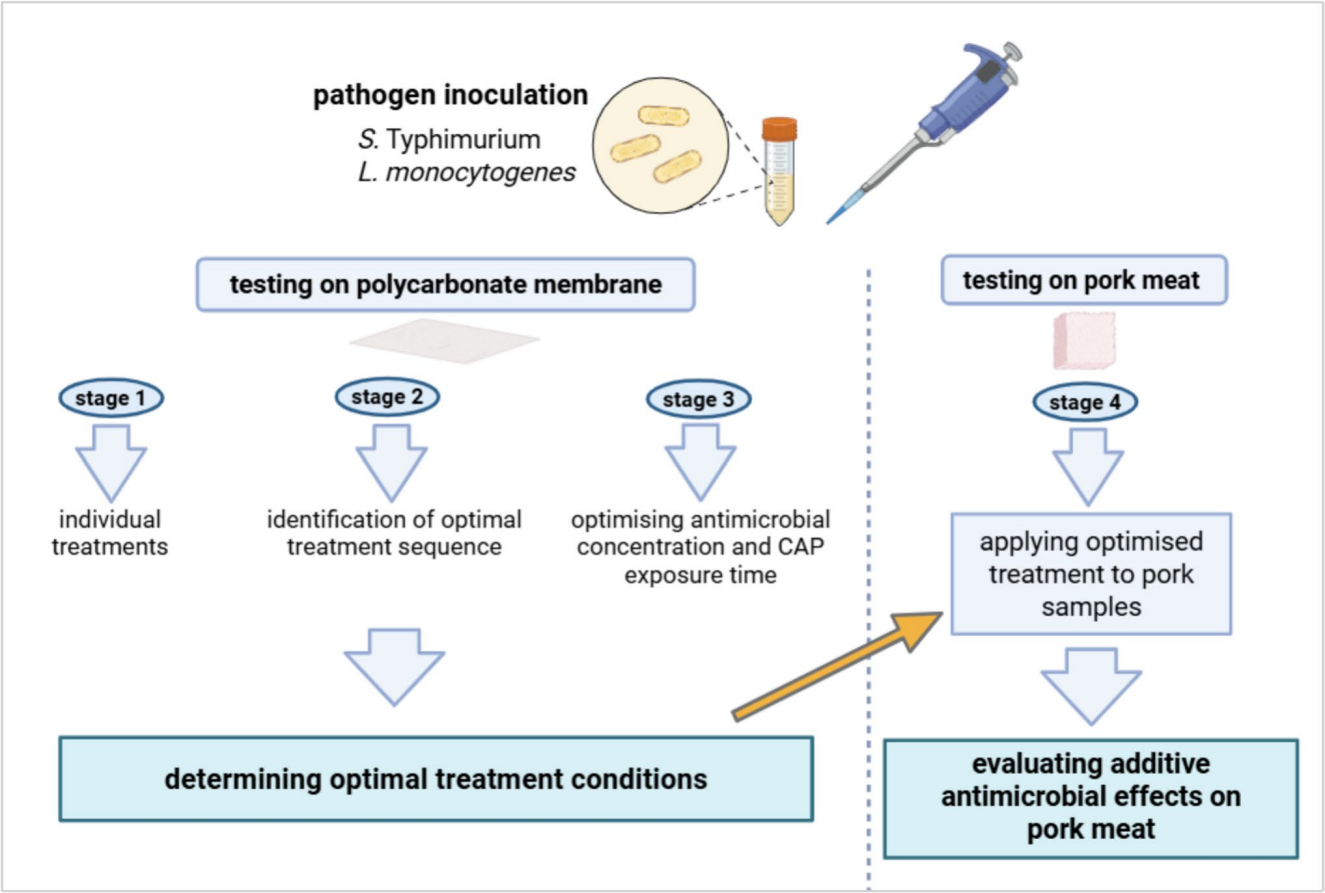
Experimental workflow illustrating CAP treatment optimisation on polycarbonate membranes (stages 1–3), followed by application of optimised conditions to pork meat (stage 4) to assess additive antimicrobial effects against *S.* Typhimurium and *L. monocytogenes*

The experimental design included multiple stages to assess the antimicrobial efficacy of CAP, both alone and in combination with antimicrobial interventions, against *Listeria monocytogenes* (WDCM 00021) and *Salmonella* Typhimurium (NCTC 112.19), with a focus on potential additive or synergistic effects. An overview of the experimental workflow is provided in Fig. 1.

Bacterial pathogens were initially prepared as described in Section “Preparation of bacterial inoculum” and then applied to polycarbonate membranes (Section "Inoculation of polycarbonate membranes with pathogenic bacteria") and pork samples (Sect. “Inoculation of pork samples with pathogenic bacteria cultures”). CAP treatment, applied at varying durations, and active compounds, tested at different concentrations, were first evaluated on polycarbonate membranes. These trials aimed to investigate the synergistic interactions between CAP exposure time and compound concentrations.

Treatment durations and compound concentrations were adjusted accordingly to ensure effectiveness in this more complex matrix. Bacterial enumeration was performed as described in Sect. “Pathogen enumeration” to quantify pathogen reduction.

### Application of bacterial inoculum and antibacterial compounds

#### Inoculation of polycarbonate membranes with pathogenic bacteria

The method was adapted from Yadav and Roopesh (2022) with minor modifications to optimise bacterial attachment and treatment conditions. Pathogen inoculum (~ 9 log CFU/mL) was applied to sterile Whatman™ polycarbonate membrane filter papers (4 cm^2^, 0.2 µm pore size; Cytiva, UK). An aliquot of 50 µL was uniformly spread across the membrane surface and incubated in a biosafety cabinet for 15 min to facilitate bacterial attachment before CAP treatment.

#### Inoculation of pork samples with pathogenic bacteria cultures

Pork samples were prepared according to the protocol of Oliinychenko et al. (2024). Briefly, pork loins were purchased from the local UK supplier (Sainsbury’s, UK) and assessed for quality by verifying pH and colour parameters following the guidelines provided by Faucitano et al. (2010). Once the pork loin was confirmed to have acceptable quality, characterised by reddish-pink, firm, and non-exudative (RFN) traits, it was then cut into uniform square pieces (10 g each) and placed in sterile Petri dishes prior to treatments.

An aliquot of 100 µL of the pathogen cell suspension (initial population ~ 9 log CFU/mL) was evenly distributed over the meat surface using a micropipette to ensure uniform inoculation. The inoculated meat samples were incubated in a laminar flow biosafety cabinet for 15 min to ensure bacterial attachment to the meat surface.

 Following attachment, the samples were subjected to CAP treatment under the specified conditions (see Section “CAP treatment of polycarbonate membrane and pork samples”). No presumptive colonies were recovered following enrichment and selective isolation; and therefore, *Salmonella* spp. and *L. monocytogenes* (using ISO 6579-1 and ISO 11290-1, respectively) were not detected in the uninoculated pork.

#### Application of antimicrobial compounds to membranes and meat samples

Antimicrobial interventions were applied at a ratio of 1:1 (v/v) between active compound and bacterial suspension to ensure uniform exposure. For each sample, an aliquot of 50 µL of bacteria and 50 µL of antibacterial compound were applied to the polycarbonate membranes and pork meat samples to account for differences in surface area and sample matrix complexity. The samples were incubated at room temperature for 30 min to facilitate interactions between the active compounds and the pathogens, following the incubation time outlined by Vihodceva et al. (2021).

Following treatment, pathogens were recovered from polycarbonate membranes and meat samples as described in Sections “Pathogen enumeration on polycarbonate membranes” and “Pathogen enumeration on pork meat”, respectively, for further analysis.

The concentrations of active compounds were determined based on their MIC/MBC values (Section “Determination of minimum inhibitory and bactericidal concentrations (MIC/MBC)”). For polycarbonate membrane tests, the active compounds were applied at their MIC/MBC concentrations. To address the complexity of the meat matrix and achieve significant pathogen reduction, antimicrobial concentrations were increased to 10 × MIC. Previous studies have shown that lower concentrations of active compounds, up to 4 × MIC, failed to achieve significant bacterial reduction on meat surfaces (Li et al. 2022a, b; Shi et al. 2024).

### CAP treatment of polycarbonate membrane and pork samples

CAP treatment was performed using a piezoelectric generator (Relyon Plasma GmbH, Germany) at an input voltage of 15 V, a frequency of 50 kHz, and ambient air as the working gas.

CAP was applied to polycarbonate membrane filter papers (4 cm^2^, 0.2 µm pore size; Cytiva, UK) prior to being inoculated with pathogens, as described in Section “Inoculation of polycarbonate membranes with pathogenic bacteria”. The polycarbonate membranes were placed in sterile Petri dishes and treated with CAP at a distance of 10 mm for durations of 0, 15, 30, and 45 s under sterile conditions. CAP was applied to pork samples inoculated with pathogens, as described in Section “Inoculation of pork samples with pathogenic bacteria cultures”. The samples were placed in sterile Petri dishes and treated with CAP at a distance of 10 mm for durations of 0, and 9 min under sterile conditions.

### Pathogen enumeration

#### Pathogen enumeration on polycarbonate membranes

Treated and untreated samples were processed following the method described by Yadav and Roopesh (2022). Briefly, samples were stored in a biosafety cabinet for 15 min before being transferred to sterile 50-mL Falcon tubes containing 4 mL of 0.1% Peptone Water (Oxoid, UK). The samples were vortexed for 60 s to detach bacterial cells from the polycarbonate membrane surface. An aliquot of 1 mL was withdrawn and serially diluted in 0.1% Peptone Water for microbial enumeration.

The two bacterial pathogens, *S.* Typhimurium and *L. monocytogenes*, were quantified by plating on pathogen-specific media under appropriate incubation conditions. For *S.* Typhimurium, enumeration was performed on Xylose-Lysine-Deoxycholate Agar (XLD; Oxoid, UK) after incubation at 37 °C for 24 h. *L. monocytogenes* was quantified on ISO-standard Chromogenic Listeria Agar (Oxoid, UK) after incubation at 37 °C for 48 h. Microbial counts were expressed as logarithmic colony-forming units per square centimetre (log CFU/cm^2^).

1 $$CFU/cm^2=number\;of\;colonies/(dilution\;factor\times volume\;plated\;(mL)\times surface\;area(cm^2))$$

#### Pathogen enumeration on pork meat

Pork samples were placed into sterile stomacher bags (Stomacher® 3500 Standard Bag, Seward Ltd., UK), and 90 mL of maximum recovery diluent (MRD; Oxoid, UK) was added to each bag. The samples were homogenised at 300 rpm for 1 min using a stomacher (400 Circulator Lab Blender; Seward Ltd., UK). The resulting homogenate was serially diluted (tenfold) in sterile MRD for bacterial enumeration.

Two bacterial pathogens, *S.* Typhimurium and *L. monocytogenes*, were enumerated on pathogen-specific media under appropriate incubation conditions. Enumeration of *S.* Typhimurium was performed on Xylose-Lysine-Deoxycholate Agar (XLD; Oxoid, UK) following incubation at 37 °C for 24 h. Enumeration of *L. monocytogenes* was conducted on Chromogenic Listeria Agar (ISO-standard; Oxoid, UK) following incubation at 37 °C for 48 h. Microbial counts were expressed as logarithmic colony-forming units per gramme (log CFU/g) of pork samples.

### Statistical analysis

The significance of differences between experimental groups was assessed using a one-way analysis of variance (ANOVA), which accounted for treatment durations, active compound concentrations, treatment combinations, and sample matrices. Before performing ANOVA, data were checked for normal distribution using the Anderson–Darling test and for homogeneity of variances using Levene’s test. All analyses were performed using Minitab software (version 21.1.0; Minitab Inc., USA). Tukey’s post hoc test was applied to identify significant differences among group means through pairwise comparisons at a significance level of *p* < 0.05. Each experiment was conducted in triplicate (*n* = 3), and results were expressed as the mean ± standard deviation (SD).

## Results and discussion

### Characterisation of citral and cinnamon nanoemulsions: PDI, zeta potential, and droplet size

Nanoemulsions are colloidal systems of oil-in-water or water-in-oil systems stabilised by emulsifiers, with droplet sizes below 200 nm (Dantas et al. 2021). A key advantage of NEs is their ability to enhance the solubility and absorption of active compounds, which has driven their increasing use as carriers for antimicrobial agents (Gupta et al. 2016). Incorporating essential oils into nanoemulsions presents a natural alternative to chemical preservatives, addressing growing concerns over antimicrobial resistance as well as increasing consumer demand for naturally derived food preservation solutions (Grand View Research 2023; Guidotti-Takeuchi et al. 2022). This section evaluates the physicochemical properties of CTR and CN nanoemulsions prepared by low-energy mixing and ultrasonication to assess their colloidal stability and potential use in food systems.

The polydispersity index (PDI) is a key indicator of droplet size uniformity in nanoemulsions, with lower values (< 0.3) indicating monodisperse systems that exhibit higher stability (Dantas et al. 2021). As shown in Table 1, the PDI values for both CTR and CN nanoemulsions were relatively low, indicating well-dispersed systems with minimal droplet aggregation. These values are in agreement with previous studies on essential oil-based nanoemulsions, where a PDI below 0.3 has been reported as optimal for ensuring stability (Pongsumpun et al. 2020). The narrow droplet size distribution suggests a well-controlled emulsification process, potentially minimising phase separation over time (Pongsumpun et al. 2020).

**Table 1:** Physicochemical properties of citral and cinnamon nanoemulsions in terms of polydispersity index (*PDI*), zeta potential (*ZP*), and mean particle diameter

Nanoemulsion	PDI	ZP, mV	Diameter size, nm
Citral	0.3 ± 0.001a	−1.9 ± 0.4a	135.9 ± 15.2a
Cinnamon	0.3 ± 0.005a	−5.2 ± 1.1b	140.7 ± 2.4a

Different letters (a and b) indicate significant differences (*p* < 0.05) between the nanoemulsions. Values are presented as mean ± standard deviation (SD). Each measurement was conducted in triplicate, with two independent experimental repetitions (*n *= 6)

Zeta potential affects nanoemulsion stability, as it determines the magnitude of electrostatic repulsion between dispersed droplets (Al-Shaiba et al. 2019). Higher ZP values correspond to stronger interparticle repulsion, thereby reducing the possibility of particle aggregation (Al-Shaiba et al. 2019). Both nanoemulsions demonstrated a high negative charge, indicating colloidal stability by promoting stronger particle repulsion (Narvekar et al. 2017).

Diameter size of nanoemulsions is another parameter that influences the stability of nanoemulsions, with optimal values typically ranging from 20 to 500 nm to ensure uniform dispersion and functional efficacy (Gupta et al. 2016). The diameter size for both CTR and CN nanoemulsions fell within this range (Table 1). Smaller droplet sizes increase the surface area available for interaction with microbial membranes, potentially enhancing antimicrobial efficacy (Wang et al. 2020).

Overall, both CTR and CN nanoemulsions exhibited physicochemical properties consistent with stable and uniform formulations. While their droplet sizes and PDI values were comparable, the more negative zeta potential of the CN suggests superior colloidal stability, which may provide a practical advantage in food preservation applications.

### MIC/MBC of antibacterial compounds against L. monocytogenes and S. Typhimurium

The antimicrobial efficacy of various compounds against *L. monocytogenes* and *S.* Typhimurium was evaluated in terms of their minimum inhibitory concentration (MIC) and minimum bactericidal concentration (MBC) (Table 2). These parameters are essential for understanding the potency of antimicrobial agents and their ability to inhibit and eliminate bacterial growth.

**Table 2:** Minimum inhibitory concentration (*MIC*) and minimum bactericidal concentration (*MBC*) of selected antimicrobial compounds against *Listeria monocytogenes* and *Salmonella* Typhimurium

Intervention		*L. monocygenes/S.* Typhimurium	
		MIC	MBC
Acids	Acetic acid	0.30%	0.60%
	Lactic acid	0.15%	0.30%
Nanoemulsions	Citral	0.25%	0.50%
	Cinnamon	0.03%	0.06%

Each measurement was conducted in triplicate, and each experiment was replicated twice (*n *= 6)

*MIC*, minimum inhibitory concentration; *MBC*, minimum bactericidal concentration

Among the tested acids, lactic acid demonstrated stronger antimicrobial activity, as evidenced by its lower MIC and MBC values for both pathogens compared to acetic acid (Table 2). This difference can be attributed to the greater ability of lactic acid to penetrate bacterial membranes and disrupt intracellular pH balance (Pangprasit et al. 2020). For the nanoemulsions, the cinnamon nanoemulsion demonstrated the highest antimicrobial activity, with the lowest MIC and MBC values for *L. monocytogenes* and *S.* Typhimurium (Table 1). These results suggest that both nanoemulsions serve as effective antimicrobial agents, with cinnamon exhibiting higher activity.

The MIC values for CN nanoemulsion (CN, MIC = 0.03%) and CTR nanoemulsion (CTR, MIC = 0.25%) are consistent with previous findings, where CN consistently exhibited higher antimicrobial potency against both Gram-positive and Gram-negative bacteria (Mandal 2011). The superior efficacy of CN compared to CTR is likely due to its greater interaction with bacterial membranes, leading to rapid inhibition of ATP synthesis and disruption of enzyme functions (Gill & Holley 2004).

### Antibacterial activity of CAP and antimicrobials on polycarbonate membranes

The current study evaluated the effectiveness of CAP alone and in combination with LA, AA, CTR and CN in reducing the bacterial levels of *S.* Typhimurium and *L. monocytogenes*. Polycarbonate membranes were used as a simplified matrix to evaluate any potential additive effects between CAP and the antimicrobials to identify the most effective combinations for application to pork meat samples. The experimental design first assessed the impact of CAP alone, followed by evaluation of treatment sequence to determine the most effective order of application (i.e., whether CAP was applied before or after the antimicrobial compounds). This was followed by an evaluation of CAP exposure time in combination with MIC or MBC levels of each compound, using the most effective treatment sequence. The analysis focused on antimicrobial activity and the contribution of each factor.

#### Antibacterial effect of individual treatments alone on polycarbonate membranes

**Fig. 2:**
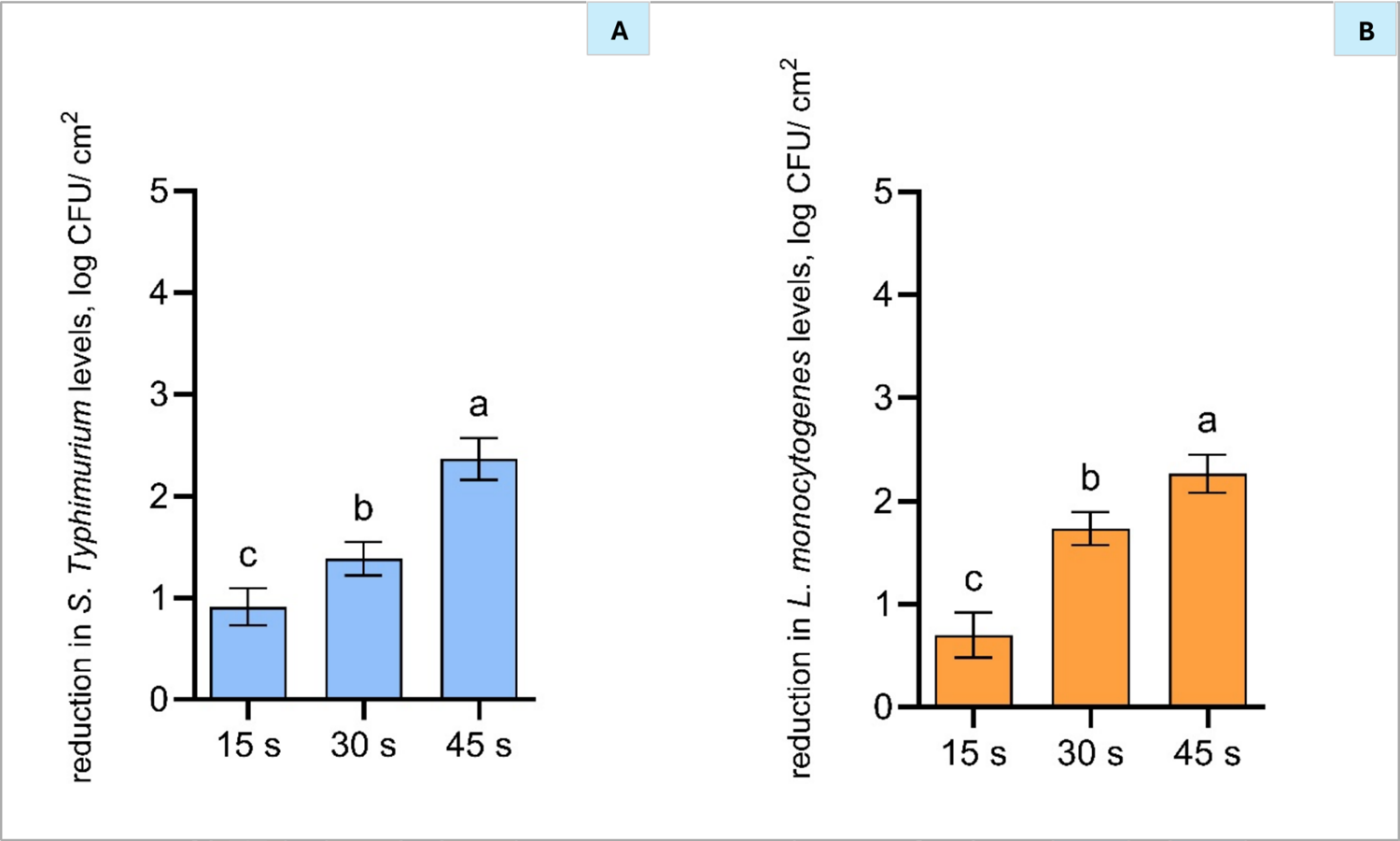
Reduction of *Salmonella* Typhimurium (**A**) and *Listeria monocytogenes* (**B**) on inoculated polycarbonate membranes following the application of cold atmospheric plasma (CAP; ambient air, 15-, 30-, and 45-s exposure). Bars represent mean ± standard deviation (SD), based on two independent experiments performed in triplicate (*n *= 6), and different letters above bars indicate significant differences between treatments (*p* < 0.05; *n* = 6)

CAP application, produced by piezoelectric discharge technology, for 15, 30, and 45 s resulted in significant reductions in bacterial levels, with *S.* Typhimurium decreasing by 0.9, 1.4, and 2.4 log CFU/cm^2^ and *L. monocytogenes* by up to 0.7, 1.7, and 2.3 log CFU/cm^2^, respectively (Fig. 2(A and B)). These results highlight CAP exposure time as a critical factor in determining its bactericidal efficacy, suggesting that optimising treatment duration may significantly enhance microbial inactivation. Our findings align with Yoo et al. (2021), who reported that CAP treatment using dielectric barrier discharge for up to 4 min significantly reduced *S. aureus* and *E. coli* in a time-dependent manner, with the greatest reductions at 3 and 4 min Yoo et al. (2021).

In the current study, there was no significant difference in CAP effectiveness between Gram-positive and Gram-negative bacteria when applied to pathogens on a filter membrane surface (Fig. 2). Consistent with our findings, Lai et al. (2016) reported no difference between bacterial Gram type and CAP efficacy when using a modified DBD system, achieving approximately 65% reduction in both Gram-negative (*Escherichia coli* and *Pseudomonas alcaligenes*) and Gram-positive (*Staphylococcus epidermidis*) bacteria. Conversely, Murali et al. (2024) observed greater CAP efficacy against Gram-positive bacteria when employing a jet CAP treatment, attributing this to higher glutathione content in the outer membrane of Gram-negative species. The variability in bacterial sensitivity to CAP (Mai-Prochnow et al. 2016) highlights the need for strategies that optimise its antimicrobial efficacy. Integrating CAP with other antimicrobial technologies has the potential to enhance pathogen inactivation while potentially allowing for milder treatment intensities. This approach might minimise undesirable quality changes in food products, making it particularly beneficial for applications in meat processing.

**Fig. 3:**
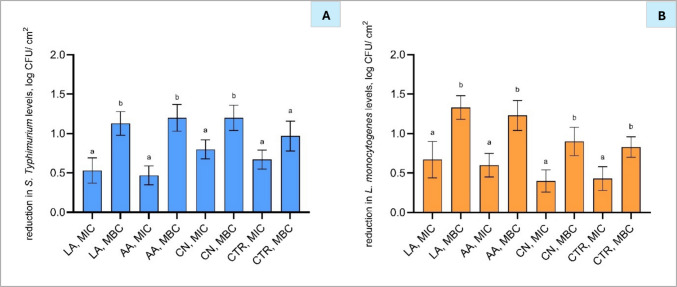
Reduction in *Salmonella* Typhimurium (**A**) and *Listeria monocytogenes* (**B**) levels (log CFU/cm^2^) on treated surfaces following application of antimicrobial compounds at their respective minimum inhibitory concentration (MIC) and minimum bactericidal concentration (MBC). Tested compounds included lactic acid (LA: MIC 0.15%, MBC 0.30%), acetic acid (AA: MIC 0.30%, MBC 0.60%), cinnamon nanoemulsion (CN: MIC 0.03%, MBC 0.06%), and citral nanoemulsion (CTR: MIC 0.25%, MBC 0.50%). Bars represent mean ± standard deviation (SD), based on two independent experiments performed in triplicate (*n* = 6), and different letters above bars indicate significant differences between treatments (*p* < 0.05; *n* = 6)

Individual treatments with LA and AA resulted in comparable reductions in *S.* Typhimurium, with concentrations of 0.15% of LA and 0.30% for AA reducing bacterial counts by 0.5 log CFU/cm^2^, and 0.30% of LA and 0.6% of AA concentrations achieving reductions of 1.2 log CFU/cm^2^ (Fig. 3(A and B)). Regarding the effect of AA and LA on *L. monocytogenes*, treatments with LA (0.15 and 0.30%) or AA (0.30 and 0.60%) resulted in reductions of 0.6 and 1.3 log CFU/cm^2^, respectively (Fig. 3(A and B)). These findings indicate comparable bactericidal effects for both acids at their respective MIC and MBC concentrations (Table 2).

Concerning the nanoemulsion treatment, individual treatments with 0.03% and 0.06% CN resulted in reductions of *S.* Typhimurium by 0.8 and 1.2 log CFU/cm^2^, and *L. monocytogenes* by 0.5 and 0.9 log CFU/cm^2^, respectively. Similarly, individual treatments with 0.25% and 0.50% CTR nanoemulsion achieved reductions of *S.* Typhimurium by 0.7 and 1.0 log CFU/cm^2^ (Fig. 3(B)) and *L. monocytogenes* by 0.4 and 0.8 log CFU/cm^2^, respectively. Previous studies have reported that Gram-negative bacteria tend to be more resistant to EOs than Gram-positive bacteria, largely due to the outer membrane of Gram-negatives, which limits the penetration of hydrophobic compounds (Nazzaro et al. 2013). However, this trend was not observed in the present study, as both pathogens showed similar MIC and MBC values to nanoemulsions (Table 2). This may be due to the use of nanoemulsions, which improved the dispersion of EOs and contact with bacterial cells, thereby enhancing antimicrobial activity (Zheng et al. 2024).

#### Determination of the effect of treatment sequence on antimicrobial activity

To determine whether the sequence of application of treatments influences the antimicrobial effectiveness, the following experiment evaluated the reduction of *S.* Typhimurium and *L. monocytogenes* after applying antimicrobial compounds either before or after CAP treatment. A treatment duration of 45 s was selected for CAP, as it had previously demonstrated significant pathogen reduction (Fig. 2) and the MBCs of the tested antimicrobials (Fig. 3). This allowed the treatment sequence to be the only variable under investigation. The antimicrobial compounds evaluated were 0.30% LA, 0.60% AA, 0.06% CN, and 0.50% CTR. Understanding the effect of treatment order is crucial for maximising the efficacy of these antibacterial treatments and supporting their potential application in food processing.

**Fig. 4:**
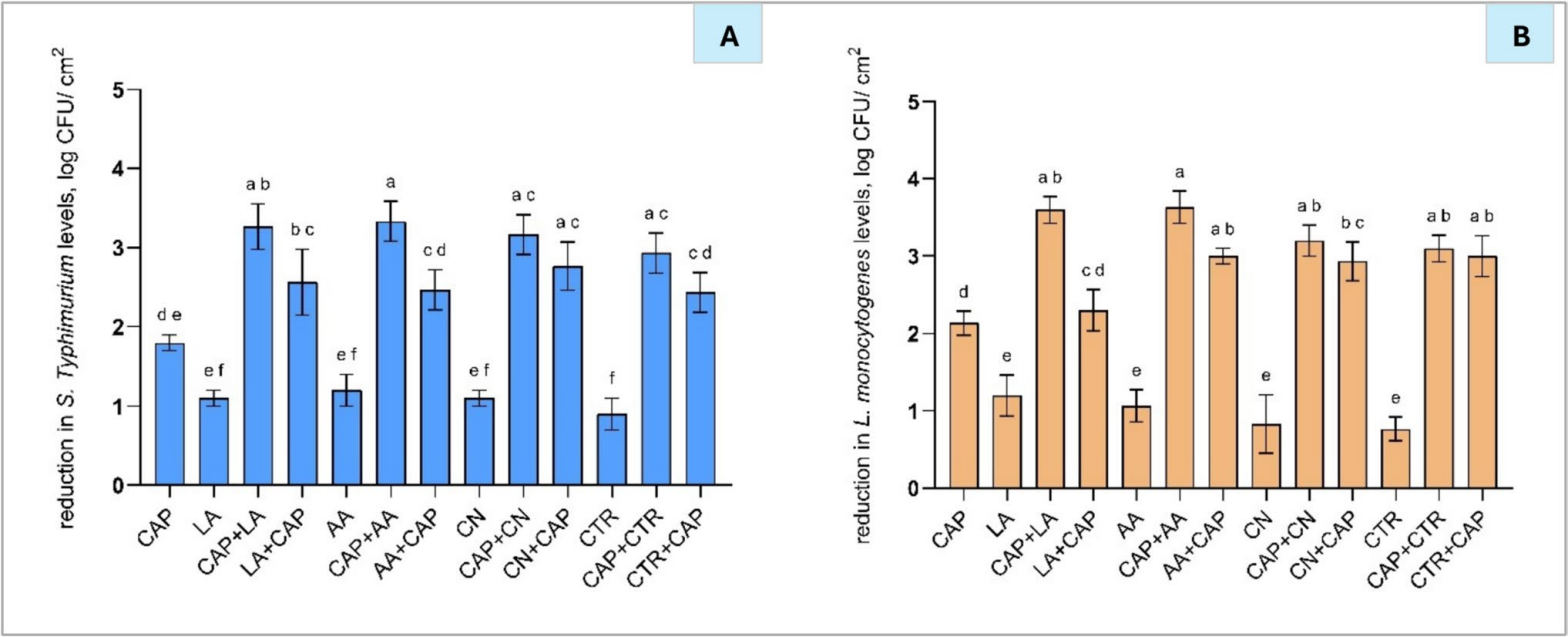
Reduction of *Salmonella* Typhimurium (**A**) and *Listeria monocytogenes* (**B**) following the application of antimicrobial compounds, either before or after cold atmospheric plasma (CAP) treatment (45 s). Bars represent mean ± standard deviation (SD), based on two independent experiments performed in triplicate (*n* = 6), and different letters above bars indicate significant differences between treatments (*p* < 0.05; *n* = 6). Abbreviations: LA, 0.30% lactic acid; AA, 0.60% acetic acid; CN, 0.06% cinnamon nanoemulsion; CTR, 0.50% citral nanoemulsion

CAP treatment alone achieved a reduction of 1.8 log CFU/cm^2^ against *S.* Typhimurium (Fig. 2(A)), while LA alone resulted in a reduction of 1.1 log CFU/cm^2^ (Fig. 3(A)). However, combining the two treatments, whether CAP was applied before or after LA, led to reductions of 3.3 and 2.6 log CFU/cm^2^, respectively, indicating an additive effect. Although CAP applied prior to LA showed a greater reduction, the difference between treatment sequences was not statistically significant (Fig. 4(A)).

Treatment with AA alone resulted in a 1.2 log CFU/cm^2^ reduction of *S.* Typhimurium, whereas applying CAP prior to AA (CAP + AA) significantly enhanced the reduction to 3.3 log CFU/cm^2^ (*p* < 0.05). However, the reverse order (AA + CAP) resulted in a significantly lower reduction of 2.4 log CFU/cm^2^. This suggests that treatment sequence is critical when using AA, with CAP treatment offering enhanced efficacy. This reduced activity in the reverse sequence may be due to CAP-induced chemical changes in AA or degradation of its active components (Prasad et al. 2023; Yoo et al. 2021). For example, Yoo et al. (2021) reported a 30% reduction in caryophyllene oxide in clove oil after CAP exposure (DBD, 15 min). These findings support the application of CAP prior to the application of antimicrobial compounds to preserve their structural integrity and functional activity.

For CN, treatment alone reduced *S.* Typhimurium by 1.1 log CFU/cm^2^. When combined with CAP (CAP + CN or CN + CAP), bacterial reductions significantly increased to ≥ 2.8 log CFU/cm^2^, with no statistically significant difference between the treatment sequences. Similarly, CTR alone achieved a reduction of 0.9 log CFU/cm^2^, while its combination with CAP resulted in enhanced reductions of ≥ 2.4 log CFU/cm^2^, again with no significant difference between sequences. These results suggest that the antimicrobial activity of nanoemulsions was not substantially influenced by the order of CAP application.

For *L. monocytogenes*, the CAP + LA treatment resulted in a significant reduction of 3.6 log CFU/cm^2^ (*p* < 0.05), compared to 2.2 log CFU/cm^2^ for CAP and 1.2 log CFU/cm^2^ for LA alone (Fig. 2(B) and 3(B), respectively). In contrast, the reverse sequence (LA + CAP) produced a significantly lower reduction of 2.3 log CFU/cm^2^, suggesting that treatment with CAP enhances the antibacterial efficacy of LA. A similar trend was observed with acetic acid, where CAP + AA treatment achieved a 3.6 log CFU/cm^2^ reduction, while AA alone and CAP alone resulted in 1.1 and 2.1 log CFU/cm^2^ reductions, respectively. The reverse sequence (AA + CAP) was significantly less effective.

For nanoemulsions, the sequence of application did not have a statistically significant effect on their antimicrobial efficacy against *L. monocytogenes* (Fig. 4(B)). CAP + CN and CN + CAP treatments showed reductions of 3.2 and 2.9 log CFU/cm^2^, respectively. Similarly, CAP + CTR and CTR + CAP achieved reductions of 3.1 and 3.0 log CFU/cm^2^, indicating that the order of application did not influence the antibacterial performance of nanoemulsions.

Overall, these results indicate an additive antibacterial effect against studied pathogens, which could be contributed to CAP-generated reactive species disrupting the bacterial membrane and facilitating deeper penetration of active compounds present in the tested antimicrobials (Prasad et al. 2023). The sequence of CAP application significantly affected the antimicrobial efficacy of organic acids (LA and AA), with treatment enhancing their effectiveness against *S.* Typhimurium and *L. monocytogenes*. However, for nanoemulsions (CN and CTR), treatment sequence did not alter antimicrobial efficacy, suggesting that their activity may not solely rely on prior bacterial membrane disruption to exert their antibacterial effects. These findings provide valuable insights for optimising food safety interventions by strategically selecting treatment sequences to maximise bacterial inactivation.

#### Antimicrobial effect of cold atmospheric plasma with lactic and acetic acid on polycarbonate membranes

**Fig. 5:**
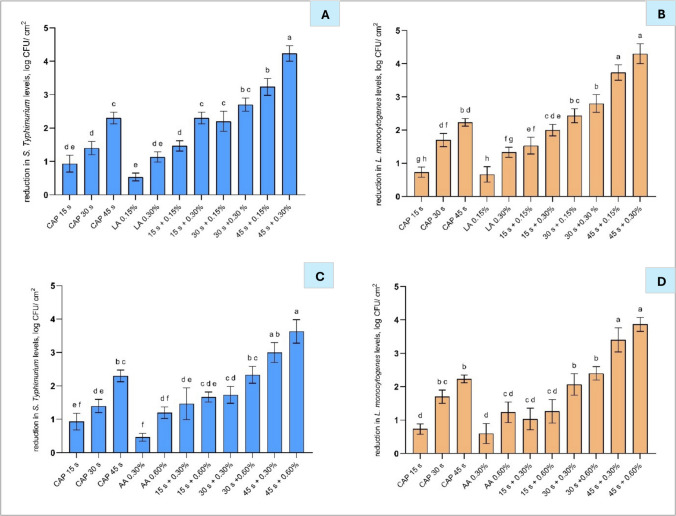
Reduction of *Salmonella* Typhimurium (**A**, **B**) and *Listeria monocytogenes* (**C**, **D**) after different CAP treatments (15, 30, 45 s), lactic acid (LA 0.15% and LA 0.30%; A and C) and acetic acid (AA 0.30% and AA 0.60%; B and D) and their combinations. Different letters indicate means differ significantly (*p* < 0.05) between treatments. Bars represent mean ± standard deviation (SD), based on two independent experiments performed in triplicate (*n* = 6), and different letters above bars indicate significant differences between treatments (*p* < 0.05; *n* = 6). Abbreviations: CAP, cold atmospheric plasma; LA, lactic acid; AA, acetic acid

The combined treatment of CAP with acids exhibited enhanced antimicrobial efficacy compared to individual treatments. When combined with LA (0.30%) or AA (0.60%), the 45-s CAP treatment showed the highest additive effects (*p *< 0.05) in reducing *S.* Typhimurium and *L. monocytogenes* (Fig. 5(A–D)), with reductions of 4.2 and 3.6 log CFU/cm^2^, respectively, compared to shorter treatment durations and the untreated bacteria, whereas the individual treatments reduced* S.* Typhimurium by 2.3 log CFU/cm^2^ following the 45-s CAP treatment, and by 1.2 log CFU/cm^2^ after the LA (0.30%) and AA (0.60%) treatments. Similarly, for *L. monocytogenes*, the 45-s CAP treatment combined with LA (0.30%) or AA (0.60%) achieved the highest reductions (*p* < 0.05) of 4.3 log CFU/cm^2^ and 3.9 log CFU/cm^2^, respectively (Fig. 5(C and D)), compared to shorter CAP treatment durations and the untreated bacteria. In comparison, the 45-s CAP treatment alone reduced *L. monocytogenes* by 2.3 log CFU/cm^2^, and LA (0.30%) and AA (0.60%) by 1.3 log CFU/cm^2^. Additive antimicrobial effects of CAP combined with organic acids can result from the combined action of distinct antimicrobial mechanisms, including acid-induced membrane disruption and oxidative damage caused by CAP-generated reactive species (Prasad et al. 2023).

Additive antimicrobial effects between CAP and organic acids have been reported in previous studies. Yadav & and Roopesh (2022) showed that treatment with lactic or gallic acid (10 mM), followed by a 30-s CAP application (DBD at 3.5 kHz), achieved a > 3.5 log CFU/cm^2^ reduction of *S.* Typhimurium on filter membranes compared to ~ 1.2 log CFU/cm^2^ by CAP alone and ~ 0.4 log CFU/cm^2^ by either acid alone. This enhanced efficacy was attributed to increased membrane permeability and oxidative stress induced by CAP, enhancing acid penetration (Xie et al. 2024; Yadav & Roopesh 2022).

However, for AA with both pathogens (Fig. 5(B and D)), and for AA with *L. monocytogenes* (Fig. 5(C)), no significant differences in bacterial reduction were observed between the MIC (0.15% for LA and 0.30% for AA) and MBC (0.15% for LA and 0.30% for AA) acid concentrations when applied with the extended 45-s CAP treatment. These findings suggest that effective bacterial inactivation can be achieved using lower concentrations of organic acids, which may minimise sensory alterations and facilitate adoption in food applications. At shorter CAP treatment times (15 and 30 s), the use of a MIC acid concentration (0.15% for LA and 0.30% for AA) resulted in no statistically significant enhancement in bacterial reduction (*p* > 0.05) compared to the individual treatments. These differences in bacterial sensitivity to acid concentration under shorter plasma exposure may be explained by the distinct modes of action: LA induces stronger cytoplasmic acidification, enhancing oxidative damage, while AA, although less effective in lowering intracellular pH, acts more directly on membrane integrity (Ji et al. 2023). In contrast, the combination of 15- and 30-s CAP treatments with LA (0.30%) and AA (0.60%) led to significantly higher reductions (*p* < 0.05) for *S.* Typhimurium and *L. monocytogenes* compared to individual treatments. Specifically, 0.30% LA combined with 15- and 30-s CAP treatments led to 2.3 and 2.7 log CFU/cm^2^ reductions of *S.* Typhimurium (Fig. 5(A)) and to 2.0 and 2.8 log CFU/cm^2^ reductions of *L. monocytogenes* (Fig. 5(C)), respectively. In comparison, 0.30% LA alone led to 1.2 log CFU/cm^2^ reduction of *S.* Typhimurium, 15- and 30-s CAP treatments to 0.9 and 1.4 log CFU/cm^2^, respectively. For *L. monocytogenes*, 0.30% LA alone led to 1.3 log CFU/cm^2^ reduction, 15- and 30-s CAP treatments reduced the bacteria by 0.7 and 1.7 log CFU/cm^2^.

#### Effect of CAP in combination with cinnamon and citral nanoemulsions on polycarbonate membranes

**Fig. 6:**
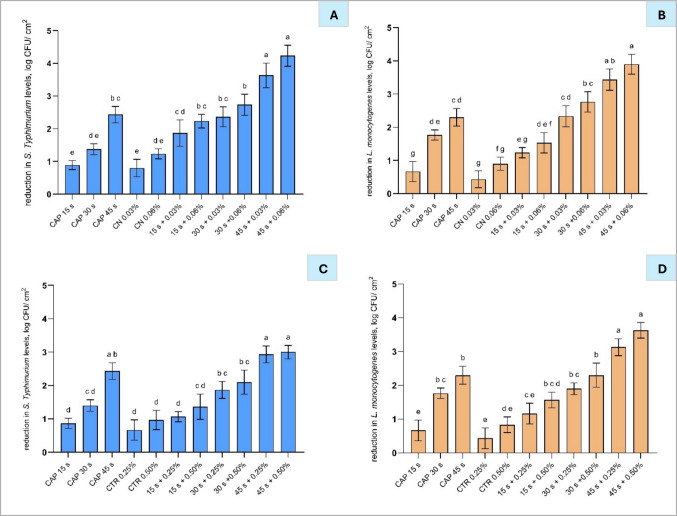
Reduction of *Salmonella* Typhimurium (**A**, **B**) and *Listeria monocytogenes* (**C**, **D**) following treatment with cold atmospheric plasma at 15, 30, and 45 s, cinnamon nanoemulsions (CN 0.03% and CN 0.06%; A and C), and citral nanoemulsions (CTR 0.25% and CTR 0.50%; B and D) and their respective combinations. Bars represent mean ± standard deviation (SD), based on two independent experiments performed in triplicate (*n* = 6), and different letters above bars indicate significant differences between treatments (*p* < 0.05; *n* = 6). Abbreviations: CAP, cold atmospheric plasma; CN, cinnamon nanoemulsion; CTR, citral nanoemulsion

Similar to LA and AA (Fig. 5), application of a lower concentration of CN (0.03%) combined with the shorter CAP treatments (15 and 30 s) did not result in significantly higher reduction (*p* > 0.05) compared to individual treatments (Fig. 6). Particularly, lower concentrations of CN and 15 and 30 s CAP treatments resulted in a reduction in *S.* Typhimurium by 1.9 and 2.4 log CFU/cm^2^ (Fig. 6(B)) and *L. monocytogenes* by 1.3 and 2.4 log CFU/cm^2^ (Fig. 6(C)) compared to treatments used alone. Despite individual treatments with 0.25% and 0.50% CTR nanoemulsion reducing *S.* Typhimurium by 0.7 and 1.0 log CFU/cm^2^ (Fig. 6(B)), respectively, the combination of CAP with CTR resulted in a significantly higher reduction compared to the individual treatments. However, the reduction was not additive, as it did not exceed the accumulative effect of the individual treatments, indicating no interaction between them, whilst the combination of 45 s CAP treatment and 0.25 and 0.50% CTR resulted in high reductions of *L. monocytogenes* by 3.1 and 3.6 log CFU/cm^2^, respectively, compared to treatments used alone. This additive antimicrobial effect can be attributed to the combined antimicrobial mechanisms of CAP and EOs when CAP-generated RONS oxidise membrane lipids, while EO components increase membrane permeability, together leading to irreversible membrane damage and improved bacterial inactivation (Prasad et al. 2023).

The results highlight the importance of optimising both CAP treatment duration and acid concentration to maximise bacterial inactivation. However, as the initial experiments were conducted using a filter membrane model, they may not fully represent the structural and compositional complexity of real food systems. Particularly, Yadav and Roopesh (2022) reported that a higher acid concentration (50 mM) was required in combination with CAPDBD to inactivate pathogens on poultry meat, likely due to the protective nature of the food matrix, which can impede acid diffusion and reduce antimicrobial efficacy. To address this, the present study also evaluated the antibacterial performance of CAP–acid combinations in pork meat, offering a more realistic assessment of their potential in food safety interventions.

### Antimicrobial efficacy of CAP and antimicrobial compounds on pork meat

#### Individual treatments on pork meat

Although essential oils can have antimicrobial properties, their application in food systems is often limited by the relatively high concentrations needed for efficacy, which can adversely affect taste and aroma (Gurtler & Garner 2022). Similarly, substantial concentrations of acids required for antimicrobial action can denature surface proteins and impart a sour taste to food (Shewail A., 2018). Therefore, investigating their application in combination with CAP offers a promising strategy to enhance the antimicrobial efficacy of antibacterial compounds without increasing their concentration, potentially minimising negative impacts on sensory and quality attributes while improving bacterial inactivation.

**Fig. 7:**
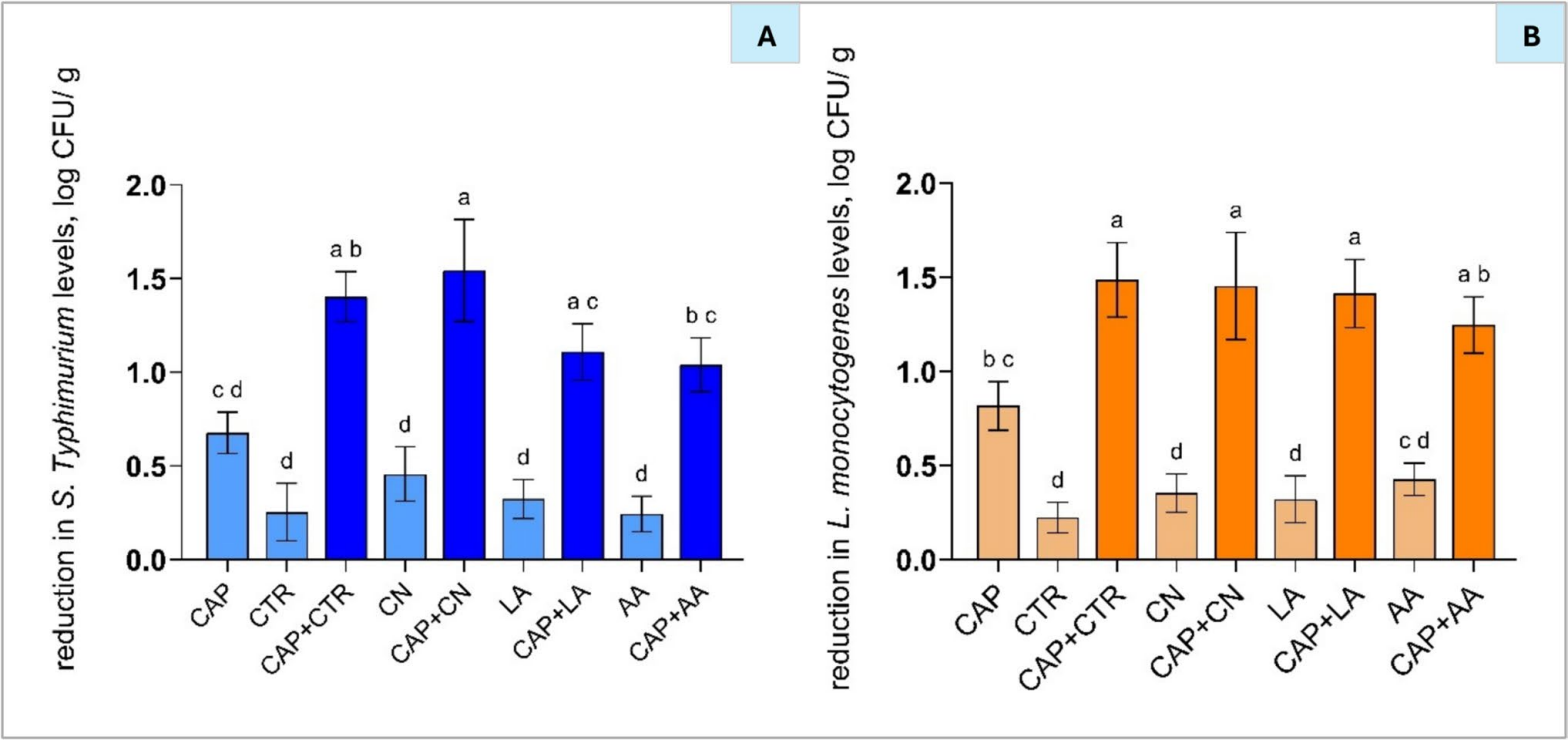
Reduction of *Salmonella* Typhimurium (**A**) and *Listeria monocytogenes* (**B**) on pork samples following treatment with antimicrobial compounds, with and without cold atmospheric plasma (CAP) exposure for 9 min. Bars represent mean ± standard deviation (SD), based on two independent experiments performed in triplicate (*n* = 6), and different letters above bars indicate significant differences between treatments (*p* < 0.05; *n* = 6). Abbreviations: LA, 1.5% lactic acid; AA, 3.0% acetic acid; CN, 0.3% cinnamon nanoemulsion; CTR, 2.5% citral nanoemulsion

In the current study, individual treatments of CTR and CN on pork meat resulted in reductions of *S.* Typhimurium and *L. monocytogenes* levels by 0.3 and 0.5 log CFU/g, and 0.2 and 0.4 log CFU/g, respectively (Fig. 7). Previous studies have demonstrated the antimicrobial effectiveness of CTR and CN nanoemulsions in meat systems. Liu et al. (2023) reported a ~ 1.0 log CFU/g reduction in total viable counts after 1 day of refrigerated storage, which increased to a ~ 2.0 log CFU/g reduction in pork treated with 5% CN after 5 days of storage, indicating that nanoemulsions exhibit enhanced antimicrobial activity with extended storage time. Similarly, Zaharioudakis et al. (2024) found that incorporating CTR and CN into edible coatings significantly reduced mesophilic bacterial populations in pork by approximately 2.0 log CFU/g during refrigerated storage. However, limitations in nanoemulsion antimicrobial efficacy have also been reported. Shi et al. (2024) observed that, despite CTR exhibiting strong antimicrobial activity in disc diffusion assays, its application in pork did not result in significant bacterial reduction at either MIC or 4 × MIC concentrations (Li et al. 2022a, b). These findings show the importance of optimising essential oil concentrations, formulations, and delivery systems in complex food matrices to achieve consistent and effective microbial control.

LA and AA demonstrated comparable reductions in *S.* Typhimurium (0.3 and 0.2 log CFU/g, respectively) and *L. monocytogenes* (0.3 and 0.4 log CFU/g, respectively), showing similar antimicrobial effectiveness to the nanoemulsions. Previous research has demonstrated the limited efficacy of organic acids when used alone for pathogen reduction on meat surfaces. Burin et al. (2014) reported that *Salmonella* spp. exposed to 0.1–1.5% LA and AA for up to 48 h continued to express acid tolerance genes, indicating bacterial survival. Similarly, Carpenter et al. (2011) observed^2011^ relatively modest reductions of *Salmonella* spp. on chicken skin and pork belly by 0.6 log CFU/cm^2^ following 2% acid washes were observed. This limited antibacterial effect is likely due to the buffering capacity of meat, which rapidly neutralises surface pH changes (Roila et al. 2022), thereby reducing the effectiveness of acids whose antimicrobial activity is primarily pH-dependent (Valle-González et al. 2020).

In the present study, 9 min of CAP treatment significantly reduced *S.* Typhimurium by 0.7 log CFU/g and *L. monocytogenes* by 0.8 log CFU/g compared to untreated samples (*p* < 0.05; Fig. 7(A–B)). This treatment duration was selected based on our previous study, in which 9 min of CAP treatment effectively inactivated spoilage bacteria without compromising the meat quality traits (Oliinychenko et al. 2024). According to previous studies, variations in CAP antibacterial efficacy can be attributed to differences in plasma treatment and generation conditions and different types of meat. For example, Choi et al. (2016) reported that corona discharge plasma achieved a 1.0 log CFU/g reduction of *L. monocytogenes* on pork, whereas Lee et al. (2016) observed a 2.7 log CFU/g reduction when applying DBD plasma to chicken meat. Additionally, Jayasena et al. (2015) found that CAP treatment using helium/oxygen as the discharge gas resulted in a 0.6 log CFU/g reduction of *L. monocytogenes*, while in-packaged CAP treatment with ambient air achieved significantly higher reductions of 2.0 log CFU/g.

#### Combined application of CAP and antimicrobials on pork meat

Unlike polycarbonate membranes, meat matrices introduce additional challenges, as fat and protein components may quench reactive species generated by CAP, thereby limiting penetration and reducing overall treatment efficacy (Jayasena et al. 2015). Particularly, Yadav a& nd Roopesh (2022) reported a ~3.5 log CFU/cm^2^ reduction of *S.* Typhimurium levels on membranes but a ~2.5 log CFU/cm^2^ reduction on poultry when combining gallic acid (50 mM) with CAP (DBD, 30 s), underscoring the impact of food matrix complexity on treatment performance.

The experiments on polycarbonate membranes (Section “Antibacterial activity of CAP and antimicrobials on polycarbonate membranes”) demonstrate that CAP treatment significantly enhances the antimicrobial efficacy of organic acids on pathogen-inoculated surfaces. Based on these preliminary results, meat samples inoculated with pathogens were subjected to CAP treatment, followed by the application of organic acids and nanoemulsion. The treatment conditions for pork meat were optimised by selecting a maximum CAP exposure time of 9 min and applying organic acids at concentrations equivalent to 10 × MIC, with the objective of maximising bacterial reduction while maintaining relevance and feasibility for food processing applications. This approach aligns with previous research showing that lower antimicrobial levels (up to 4 × MIC) were insufficient in meat (Li et al. 2022a, b), while 9 min of CAP treatment showed effective bacterial inactivation without compromising meat quality (Oliinychenko et al. 2025). Overall, these parameters were identified as the most promising approach for enhancing microbial safety in pork.

The combination of cold plasma with both organic acids and nanoemulsions significantly enhanced antimicrobial efficacy compared to individual treatments, highlighting its potential to address limitations associated with the relatively high antibacterial concentrations (Section “Preparation of lactic and acetic acid solutions”), which can adversely impact food quality (Gurtler & Garner 2022; Shewail A., 2018). Particularly, organic acids (LA and AA) alone showed limited reductions of a ~ 0.4 log CFU/g reduction against the tested pathogens, whilst their combination with CAP resulted in significantly greater bacterial reduction. Particularly, CAP + LA treatment reduced *S.* Typhimurium and *L. monocytogenes* by 1.1 and 1.4 log CFU/g, respectively, and CAP + AA treatment by 1.0 and 1.3 log CFU/g, respectively. Combinations of CAP with nanoemulsions similarly demonstrated high reductions compared to acids, suggesting a strong additive effect. Specifically, CAP + CN treatment achieved reductions of ~ 1.5 log CFU/g for *S.* Typhimurium and *L. monocytogenes* levels, while CAP + CTR treatment led to similar reductions of ~ 1.4 log CFU/g for both pathogen levels, respectively (Fig. 7(A–B)), indicating comparable effectiveness of NEs when applied at their MIC × 10 concentrations and combined with CAP.

In previous studies combining CAP with oil-based nanoemulsions, Sahebkar et al. (2020) reported additive antibacterial effects. Specifically, CAP treatment (DBD, 10 min) applied to chicken marinated in a blend of EOs led to a ~ 3.0 log CFU/g reduction of *Staphylococcus aureus* and *Escherichia coli* levels. Importantly, 14-day refrigerated storage was shown to enhance the antimicrobial activity of nanoemulsions combined with CAP in the study by Sahebkar et al. (2020). Similarly, Liu et al. (2023) reported increased efficacy of nanoemulsions applied alone, supporting the idea that their antimicrobial activity may improve over time due to prolonged release and sustained interaction with bacterial cells. The comparatively lower additive effect observed for the CAP + NE treatment (~ 1.5 log CFU/g reduction for both pathogens) in the present study may be attributed to differences in the type of meat used, variations in CAP generation conditions, and the absence of a storage period, during which antimicrobials may have exerted greater impact due to extended contact time with pathogens.

In the context of acid-based treatments and CAP, Kang et al. (2022) reported an approximately 1.0 log CFU/g reduction of *S.* Typhimurium levels on chicken following treatment with 0.8% acetic acid pre-activated by CAP using DBD for 30 min. The enhanced antimicrobial efficacy of CAP-activated acetic acid was attributed to the generation of reactive oxygen species (ROS), which contributed to a decrease in pH and enhanced the overall antibacterial activity of the solution (Heng et al. 2022; Kang et al. 2022). Although the reduction observed in the study by Kang et al. (2022) was comparable to the present findings, CAP treatment in the current study was intentionally applied prior to acid treatment, as preliminary trials showed this sequence to be the most effective (Section “Determination of the effect of treatment sequence on antimicrobial activity”). Additionally, applying CAP first helped to minimise acidification of the residual organic acids, which can negatively affect meat quality. For example, Manzoor et al. (2020) and Pipek et al. (2005) reported significant colour deterioration in beef and pork, respectively, following treatment with 6% and 2% LA solutions (pH ~ 2.2–2.8). Therefore, the treatment sequence applied in the current research may offer a more suitable approach for achieving effective microbial inactivation when applied to meat.

Overall, this study demonstrates that CAP combined with nanoemulsions and acids significantly enhances bacterial inactivation in pork meat, offering a promising approach to improve meat safety while reducing the need for high concentrations of individual antimicrobials.

## Conclusions

This study demonstrated that PDD-CAP, applied for 15–45 s on polycarbonate membranes and 9 min on pork, was effective against *Salmonella* Typhimurium and *Listeria monocytogenes*, and that its combination with lactic acid (0.15–0.30%), acetic acid (0.30–0.60%), or antimicrobial nanoemulsions (cinnamon 0.03–0.06%; citral 0.25–0.50%) further enhanced antimicrobial efficacy on both matrices.

On polycarbonate membranes, PDD-CAP alone achieved time-dependent reductions of up to 2.4 log CFU/cm^2^ for *S.* Typhimurium and 2.3 log CFU/cm^2^ for *L. monocytogenes*, whereas combining PDD-CAP with lactic or acetic acid at MIC/MBC produced higher reductions of approximately 3.6 log CFU/cm^2^.

The sequence of application was critical for organic acids on membranes, with PDD-CAP followed by lactic or acetic acid (~ 3.6 log CFU/cm^2^ reduction) being more effective than acids applied before PDD-CAP (~ 2.3 log CFU/cm^2^), consistent with PDD-CAP-induced membrane damage facilitating acid penetration; in contrast, the treatment sequence did not influence the efficacy of nanoemulsions at MIC/MBC.

On pork, PDD-CAP treatment for 9 min combined with organic acids or nanoemulsions at 10× MIC produced additive effects, improving pathogen inactivation by around 1.5 log CFU/g compared with either PDD-CAP alone or antimicrobials alone under the same conditions, thereby confirming the applicability of the most effective sequences identified on membranes to a meat matrix.

Overall, these findings support the integration of CAP with antimicrobial compounds as a sustainable, non-thermal strategy for pathogen control in the meat industry. This approach aligns with consumer and industry preferences for minimally processed meat products with extended shelf life while reducing reliance solely on preservatives. Future research should focus on optimising CAP treatment parameters and further evaluating the effects of combined CAP–antimicrobial applications on sensory attributes such as colour, texture, and flavour to ensure meat quality and consumer acceptability in large-scale applications.

## Data Availability

The data that support the findings of this study are not openly available due to reasons of sensitivity and are available from the corresponding author upon reasonable request.
